# General Decidability Results for Asynchronous Shared-Memory Programs: Higher-Order and Beyond

**DOI:** 10.1007/978-3-030-72016-2_24

**Published:** 2021-03-01

**Authors:** Rupak Majumdar, Ramanathan S. Thinniyam, Georg Zetzsche

**Affiliations:** 8 Eindhoven University of Technology, Eindhoven, The Netherlands; 9 Aalborg University, Aalborg East, Denmark; Max Planck Institute for Software Systems (MPI-SWS), Kaiserslautern, Germany

**Keywords:** Higher-order asynchronous programs, Decidability

## Abstract

The model of asynchronous programming arises in many contexts, from low-level systems software to high-level web programming. We take a language-theoretic perspective and show general decidability and undecidability results for asynchronous programs that capture all known results as well as show decidability of new and important classes. As a main consequence, we show decidability of safety, termination and boundedness verification for *higher-order* asynchronous programs—such as OCaml programs using Lwt—and undecidability of liveness verification already for order-2 asynchronous programs. We show that under mild assumptions, surprisingly, safety and termination verification of asynchronous programs with handlers from a language class are decidable *iff* emptiness is decidable for the underlying language class. Moreover, we show that configuration reachability and liveness (fair termination) verification are equivalent, and decidability of these problems implies decidability of the well-known “equal-letters” problem on languages. Our results close the decidability frontier for asynchronous programs.

## 1 Introduction

*Asynchronous programming* is a common way to manage concurrent requests in a system. In this style of programming, rather than waiting for a time-consuming operation to complete, the programmer can make *asynchronous* procedure calls which are stored in a *task buffer* pending later execution. Each asynchronous procedure, or *handler*, is a sequential program. When run, it can change the *global shared state* of the program, make internal synchronous procedure calls, and post further instances of handlers to the task buffer. A scheduler repeatedly and non-deterministically picks pending handler instances from the task buffer and executes their code *atomically* to completion. Asynchronous programs appear in many domains, such as operating system kernel code, web programming,

* This research was sponsored in part by the Deutsche Forschungsgemeinschaft project 389792660 TRR 248–CPEC and by the European Research Council under the Grant Agreement 610150 (ERC Synergy Grant ImPACT).

or user applications on mobile platforms. This style of programming is supported natively or through libraries for most programming environments. The interleaving of different handlers hides latencies of long-running operations: the program can process a different handler while waiting for an external operation to finish. However, asynchronous scheduling of tasks introduces non-determinism in the system, making it difficult to reason about correctness.

An asynchronous program is *finite-data* if all program variables range over finite domains. Finite-data programs are still infinite state transition systems: the task buffer can contain an unbounded number of pending instances and the sequential machine implementing an individual handler can have unboundedly large state (e.g., if the handler is given as a recursive program, the stack can grow unboundedly). Nevertheless, verification problems for finite-data programs have been shown to be decidable for several kinds of handlers [12,30,20,6]. Several algorithmic approaches have been studied, which tailor to (i) the kinds of permitted handler programs and (ii) the properties that are checked.

**State of the art** We briefly survey the existing approaches and what is known about the decidability frontier. The *Parikh approach* applies to (first-order) recursive handler programs. Here, the decision problems for asynchronous programs are reduced to decision problems over Petri nets [12]. The key insight is that since handlers are executed atomically, the order in which a handler posts tasks to the buffer is irrelevant. Therefore, instead of considering the sequential order of posted tasks along an execution, one can equivalently consider its Parikh image. Thus, when handlers are given pushdown systems, the behaviors of an asynchronous program can be represented by a (polynomial sized) Petri net. Using the Parikh approach, safety (formulated as reachability of a global state), termination (whether all executions terminate), and boundedness (whether there is an a priori upper bound on the task buffer) are all decidable for asynchronous programs with recursive handlers, by reduction to corresponding problems on Petri nets [30,12]. Configuration reachability (reachability of a specific global state and task buffer configuration), fair termination (termination under a fair scheduler), and fair non-starvation (every pending handler instance is eventually executed) are also decidable, by separate ad hoc reductions to Petri net reachability [12]. A "reverse reduction" shows that Petri nets can be simulated by polynomial-sized asynchronous programs (already with finite-data handlers).

In the *downclosure approach*, one replaces each handler with a finite-data program that is equivalent up to "losing" handlers in the task buffer. Of course, this requires that one can compute equivalent finite-data programs for given handler programs. This has been applied to checking safety for recursive handler programs [3]. Finally, a bespoke *rank-based approach* has been applied to checking safety when handlers can perform restricted higher-order recursion [6].

**Contribution** Instead of studying individual kinds of handler programs, we consider asynchronous programs in a general language-theoretic framework. The class of handler programs is given as a language class $\mathcal{C}$: An asynchronous program over a language class $\mathcal{C}$ is one where each handler defines a language from $\mathcal{C}$ over the alphabet of handler names, as well as a transformer over the global state. This view leads to general results: we can obtain simple characterizations of which classes of handler programs permit decidability. For example, we do not need the technical assumptions of computability of equivalent finite-data programs from the Parikh and the downclosure approach.

Our first result shows that, under a mild language-theoretic assumption, safety and termination are decidable if and only if the underlying language class $\mathcal{C}$ has decidable emptiness problem.[1] Similarly, we show that boundedness is decidable iff *finiteness* is decidable for the language class $\mathcal{C}$. These results are the best possible: decidability of emptiness (resp., finiteness) is a requirement for safety and termination verification already for verifying the safety or termination (resp., boundedness) of one *sequential* handler call. As corollaries, we get new decidability results for all these problems for asynchronous programs over *higher-order recursion schemes*, which form the language-theoretic basis for programming in higher-order functional languages such as OCaml [21,28], as well as other language classes (lossy channel languages, Petri net languages, etc.).

Second, we show that configuration reachability, fair termination, and fair starvation are mutually reducible; thus, decidability of any one of them implies decidability of all of them. We also show decidability of these problems implies the decidability of a well-known combinatorial problem on languages: given a language over the alphabet $\{\mathtt{a}, \mathtt{b}\}$, decide if it contains a word with an equal number of $\mathtt{a}$s and $\mathtt{b}$s. Viewed contrapositively, we conclude that all these decision problems are undecidable already for asynchronous programs over order-2 pushdown languages, since the equal-letters problem is undecidable for this class.

Together, our results "close" the decidability frontier for asynchronous programs, by demonstrating reducibilities between decision problems heretofore studied separately and connecting decision problems on asynchronous programs with decision problems on the underlying language classes of their handlers.

While our algorithms do not assume that downclosures are effectively computable, we use downclosures to prove their correctness. We show that safety, termination, and boundedness problems are invariant under taking downclosures of runs; this corresponds to taking downclosures of the languages of handlers.

The observation that safety, termination, and boundedness depend only on the downclosure suggests a possible route to implementation. If there is an effective procedure to compute the downclosure for class $\mathcal{C}$, then a direct verification algorithm would replace all handlers by their (regular) downclosures, and invoke existing decision procedures for this case. Thus, we get a direct algorithm based on downclosure constructions for higher order recursion schemes, using the string of celebrated recent results on effectively computing the downclosure of *word schemes* [33,15,7].

We find our general decidability result for asynchronous programs to be surprising. Already for regular languages, the complexity of safety verification jumps

[1] The "mild language-theoretic assumption" is that the class of languages forms an effective full trio: it is closed under intersections with regular languages, homomorphisms, and inverse homomorphisms. Many language classes studied in formal language theory and verification satisfy these conditions.

from NL (NFA emptiness) to EXPSPACE (Petri net coverability): asynchronous programs are far more expressive than individual handler languages. It is therefore surprising that safety and termination verification remains decidable whenever it is decidable for individual handler languages.

Full proofs of our results are available here [25].

## 2 Preliminaries

*Basic Definitions* We assume familiarity with basic definitions of automata theory (see, e.g., [18,31]). The projection of word $w$ onto some alphabet $\Sigma'$, written $\mathrm{Proj}_{\Sigma'}(w)$, is the word obtained by erasing from $w$ each symbol which does not belong to $\Sigma'$. For a language $L$, define $\mathrm{Proj}_{\Sigma'}(L) = \{\mathrm{Proj}_{\Sigma'}(w) \mid w \in L\}$. The *subword* order $\sqsubseteq$ on $\Sigma^*$ is defined as $w \sqsubseteq w'$ for $w, w' \in \Sigma^*$ if $w$ can be obtained from $w'$ by deleting some letters from $w'$. For example, $abba \sqsubseteq bababa$ but $abba \not\sqsubseteq baaba$. The *downclosure* $\downarrow w$ with respect to the subword order of a word $w \in \Sigma^*$ is defined as $\downarrow w := \{w' \in \Sigma^* \mid w' \sqsubseteq w\}$. The downclosure $\downarrow L$ of a language $L \subseteq \Sigma^*$ is given by $\downarrow L := \{w' \in \Sigma^* \mid \exists w \in L \colon w' \sqsubseteq w\}$. Recall that the downclosure $\downarrow L$ of any language $L$ is a regular language [17].

A *multiset* $\mathbf{m} \colon \Sigma \to \mathbb{N}$ over $\Sigma$ maps each symbol of $\Sigma$ to a natural number. Let $\mathbb{M}[\Sigma]$ be the set of all multisets over $\Sigma$. We treat sets as a special case of multisets where each element is mapped onto 0 or 1. As an example, we write $\mathbf{m} = [\![\mathtt{a}, \mathtt{a}, \mathtt{c}]\!]$ for the multiset $\mathbf{m} \in \mathbb{M}[\{\mathtt{a}, \mathtt{b}, \mathtt{c}, \mathtt{d}\}]$ such that $\mathbf{m}(\mathtt{a}) = 2$, $\mathbf{m}(\mathtt{b}) = \mathbf{m}(\mathtt{d}) = 0$, and $\mathbf{m}(\mathtt{c}) = 1$. We also write $|\mathbf{m}| = \sum_{\sigma \in \Sigma} \mathbf{m}(\sigma)$.

Given two multisets $\mathbf{m}, \mathbf{m}' \in \mathbb{M}[\Sigma]$ we define the multiset $\mathbf{m} \oplus \mathbf{m}' \in \mathbb{M}[\Sigma]$ for which, for all $\mathtt{a} \in \Sigma$, we have $(\mathbf{m} \oplus \mathbf{m}')(\mathtt{a}) = \mathbf{m}(\mathtt{a}) + \mathbf{m}'(\mathtt{a})$. We also define the natural order $\preceq$ on $\mathbb{M}[\Sigma]$ as follows: $\mathbf{m} \preceq \mathbf{m}'$ iff there exists $\mathbf{m}^{\Delta} \in \mathbb{M}[\Sigma]$ such that $\mathbf{m} \oplus \mathbf{m}^{\Delta} = \mathbf{m}'$. We also define $\mathbf{m}' \ominus \mathbf{m}$ for $\mathbf{m} \preceq \mathbf{m}'$ analogously: for all $\mathtt{a} \in \Sigma$, we have $(\mathbf{m} \ominus \mathbf{m}')(\mathtt{a}) = \mathbf{m}(\mathtt{a}) - \mathbf{m}'(\mathtt{a})$. For $\Sigma \subseteq \Sigma'$ we regard $\mathbf{m} \in \mathbb{M}[\Sigma]$ as a multiset of $\mathbb{M}[\Sigma']$ where undefined values are sent to 0.

*Language Classes and Full Trios* A *language class* is a collection of languages, together with some finite representation. Examples are the regular (e.g. represented by finite automata) or the context-free languages (e.g. represented by pushdown automata or PDA). A relatively weak and reasonable assumption on a language class is that it is a *full trio*, that is, it is closed under each of the following operations: taking intersection with a regular language, taking homomorphic images, and taking inverse homomorphic images. Equivalently, a language class is a full trio iff it is closed under *rational transductions* [5].

We assume that all full trios $\mathcal{C}$ considered in this paper are *effective*: Given a language $L$ from $\mathcal{C}$, a regular language $R$, and a homomorphism $h$, we can compute a representation of the languages $L \cap R$, $h(L)$, and $h^{-1}(L)$ in $\mathcal{C}$.

Many classes of languages studied in formal language theory form effective full trios. Examples include the regular and the context-free languages [18], the indexed languages [2,10], the languages of higher-order pushdown automata [26], higher-order recursion schemes (HORS) [16,9], Petri nets [14,19], and lossy channel systems (see Section 4.1). (While HORS are usually viewed as representing a tree or collection of trees, one can also view them as representing a word language, as we explain in Section 5.)

Informally, a language class defined by non-deterministic devices with a finite-state control that allows $\varepsilon$-transitions and imposes no restriction between input letter and performed configuration changes (such as non-deterministic pushdown automata) is always a full trio: The three operations above can be realized by simple modifications of the finite-state control. The deterministic context-free languages are a class that is *not* a full trio.

*Asynchronous Programs: A Language-Theoretic View* We use a language-theoretic model for asynchronous shared-memory programs.

**Definition 1.** *Let $\mathcal{C}$ be an (effective) full trio. An* asynchronous program *(*AP*) over $\mathcal{C}$ is a tuple $\mathfrak{P} = (D, \Sigma, (L_c)_{c\in\mathfrak{C}}, d_0, \mathbf{m}_0)$, where $D$ is a finite set of* global states*, $\Sigma$ is an alphabet of* handler names*, $(L_c)_{c\in\mathfrak{C}}$ is a family of languages from $\mathcal{C}$, one for each $c \in \mathfrak{C}$ where $\mathfrak{C} = D \times \Sigma \times D$ is the set of contexts, $d_0 \in D$ is the* initial state*, and $\mathbf{m}_0 \in \mathbb{M}[\Sigma]$ is a multiset of* initial pending handler instances.

*A* configuration $(d, \mathbf{m}) \in D \times \mathbb{M}[\Sigma]$ *of $\mathfrak{P}$ consists of a global state $d$ and a multiset $\mathbf{m}$ of pending handler instances. For a configuration $c$, we write $c.d$ and $c.\mathbf{m}$ for the global state and the multiset in the configuration respectively. The* initial *configuration $c_0$ of $\mathfrak{P}$ is given by $c_0.d = d_0$ and $c_0.\mathbf{m} = \mathbf{m}_0$. The semantics of $\mathfrak{P}$ is given as a labeled transition system over the set of configurations, with the transition relation $\xrightarrow{\sigma} \subseteq (D \times \mathbb{M}[\Sigma]) \times (D \times \mathbb{M}[\Sigma])$ given by*

$$(d, \mathbf{m} \oplus [\![\sigma]\!]) \xrightarrow{\sigma} (d', \mathbf{m} \oplus \mathbf{m}') \quad \textit{iff} \quad \exists w \in L_{d\sigma d'} : \mathsf{Parikh}(w) = \mathbf{m}'$$

*We use $\rightarrow^*$ for the reflexive transitive closure of the transition relation. A configuration $c$ is said to be* reachable *in $\mathfrak{P}$ if $(d_0, \mathbf{m}_0) \rightarrow^* c$.*

Intuitively, the set $\Sigma$ of handler names specifies a finite set of procedures that can be invoked asynchronously. The shared state takes values in $D$. When a handler is called asynchronously, it gets added to a bag of pending handler calls (the multiset $\mathbf{m}$ in a configuration). The language $L_{d\sigma d'}$ captures the effect of executing an instance of $\sigma$ starting from the global state $d$, such that on termination, the global state is $d'$. Each word $w \in L_{d\sigma d'}$ captures a possible sequence of handlers posted during the execution.

Suppose the current configuration is $(d, \mathbf{m})$. A non-deterministic scheduler picks one of the outstanding handlers $\sigma \in \mathbf{m}$ and executes it. Executing $\sigma$ corresponds to picking one of the languages $L_{d\sigma d'}$ and some word $w \in L_{d\sigma d'}$. Upon execution of $\sigma$, the new configuration has global state $d'$ and the new bag of pending calls is obtained by taking $\mathbf{m}$, removing an instance of $\sigma$ from it, and adding the Parikh image of $w$ to it. This reflects the current set of pending handler calls—the old ones (minus an instance of $\sigma$) together with the new ones added by executing $\sigma$. Note that a handler is executed atomically; thus, we atomically update the global state and the effect of executing the handler.

Let us see some examples of asynchronous programs. It is convenient to present these examples in a programming language syntax, and to allow each

```
1  global var turn = ref 0 and x = ref 0;
2  let rec s1 () = if * then begin post a; s1(); post b end
3  let rec s2 () = if * then begin post a; s2(); post b end else post b
4  let a () = if !turn = 0 then begin turn := 1; x := !x + 1 end else post a
5  let b () = if !turn = 1 then begin turn := 0; x := !x - 1 end else post b
6
7  let s3 () = post s3; post s3
8
9  global var t = ref 0;
10 let c () = if !t = 0 then t := 1 else post c
11 let d () = if !t = 1 then t := 2 else post d
12 let f () = if !t = 2 then t := 0 else post f
13
14 let cc x = post c; x
15 let dd x = post d; x
16 let ff x = post f; x
17 let id x = x
18 let h g y = cc (g (dd y))
19 let rec produce g x = if * then produce (h g) (ff x) else g x
20 let s4 () = produce id  ()
```

**Fig. 1.** Examples of asynchronous programs handler to have *internal actions* that perform local tests and updates to the global state. As we describe informally below, and formally in the full version, when $\mathcal{C}$ is a full trio, internal actions can be "compiled away" by taking an intersection with a regular language of internal actions and projecting the internal actions away. Thus, we use our simpler model throughout.

*Examples* Figure 1 shows some simple examples of asynchronous programs in an OCaml-like syntax. Consider first the asynchronous program in lines 1–5. The alphabet of handlers is `s1`, `s2`, `a`, and `b`. The global states correspond to possible valuations to the global variables `turn` and `x`; assuming `turn` is a Boolean and `x` takes values in $\mathbb{N}$, we have that $D = \{0, 1\} \times \{0, 1, \omega\}$, where $\omega$ abstracts all values other than $\{0, 1\}$. Since `s1` and `s2` do not touch any variables, for $d, d' \in D$, we have $L_{d,\texttt{s1},d} = \{\texttt{a}^n\texttt{b}^n \mid n \geq 0\}$, $L_{d,\texttt{s2},d} = \{\texttt{a}^n\texttt{b}^{n+1} \mid n \geq 0\}$, and $L_{d,\texttt{s1},d'} = L_{d,\texttt{s2},d'} = \emptyset$ if $d' \neq d$.

For the languages corresponding to `a` and `b`, we use syntactic sugar in the form of *internal actions*; these are local tests and updates to the global state. For our example, we have, e.g., $L_{(0,0),\texttt{a},(1,1)} = \{\varepsilon\}$, $L_{(1,x),\texttt{a},(1,x)} = \{\texttt{a}\}$ for all values of $x$, and similarly for `b`. The meaning is that, starting from a global state $(0, 0)$, executing the handler will lead to the global state $(1, 1)$ and no handlers will be posted, whereas starting from a global state in which `turn` is 1, executing the handler will keep the global state unchanged but post an instance of `a`. Note that all the languages are context-free.

Consider an execution of the program from the initial configuration $((0, 0), [\![\texttt{s1}]\!])$. The execution of `s1` puts $n$ `a`s and $n$ `b`s into the bag, for some $n \geq 0$. The global variable `turn` is used to ensure that the handlers `a` and `b` alternately update `x`. When `turn` is 0, the handler for `a` increments `x` and sets `turn` to 1, otherwise it re-posts itself for a future execution. Likewise, when `turn` is 1, the handler for `b` decrements `x` and sets `turn` back to 0, otherwise it re-posts itself for a future execution. As a result, the variable `x` never grows beyond 1. Thus, the program satisfies the *safety* property that no execution sets `x` to $\omega$.

It is possible that the execution goes on forever: for example, if `s1` posts an `a` and a `b`, and thereafter only `b` is chosen by the scheduler. This is not an "interesting" infinite execution as it is not fair to the pending `a`. In the case of a fair scheduler, which eventually always picks an instance of every pending task, the program terminates: eventually all the `a`s and `b`s are consumed when they are scheduled in alternation. However, if instead we started with $[\![\mathtt{s2}]\!]$, the program will not terminate even under a fair scheduler: the last remaining `b` will not be paired and will keep executing and re-posting itself forever.

Now consider the execution of `s3`. It has an infinite fair run, where the scheduler picks an instance of `s3` at each step. However, the number of pending instances grows without bound. We shall study the *boundedness problem*, which checks if the bag can become unbounded along some run. We also study a stronger notion of fair termination, called *fair non-starvation*, which asks that every *instance* of a posted handler is executed under any fair scheduler. The execution of `s3` is indeed fair, but there can be a specific instance of `s3` that is never picked: we say `s3` can *starve* an instance.

The program in lines 9–20 is *higher-order* (`produce` and `h` take functions as arguments). The language of `s4` is the set $\{\mathtt{c}^n\mathtt{d}^n\mathtt{f}^n \mid n \geq 0\}$, that is, it posts an equal number of `c`s, `d`s, and `f`s. It is an indexed language; we shall see (Section 5) how this and other higher-order programs can be represented using higher-order recursion schemes (HORS). Note the OCaml types of `produce` : $(\mathtt{o} \to \mathtt{o}) \to \mathtt{o} \to \mathtt{o}$ and `h` : $(\mathtt{o} \to \mathtt{o}) \to \mathtt{o} \to \mathtt{o}$ are higher-order.

The program is similar to the first: the handlers `c`, `d`, and `f` execute in "round robin" fashion using the global state `t` to find their turns. Again, we use internal actions to update the global state for readability. We ask the same decision questions as before: does the program ever reach a specific global state and does the program have an infinite (fair) run? We shall see later that safety and termination questions remain decidable, whereas fair termination does not.

## 3 Decision Problems on Asynchronous Programs

We now describe decision problems on runs of asynchronous programs.

*Runs, preruns, and downclosures* A *prerun* of an AP $\mathfrak{P} = (D, \Sigma, (L_c)_{c\in\mathfrak{C}}, d_0, \mathbf{m}_0)$ is a finite or infinite sequence $\rho = (e_0, \mathbf{n}_0), \sigma_1, (e_1, \mathbf{n}_1), \sigma_2, \ldots$ of alternating elements of tuples $(e_i, \mathbf{n}_i) \in D \times \mathbb{M}[\Sigma]$ and symbols $\sigma_i \in \Sigma$. The set of preruns of $\mathfrak{P}$ will be denoted $\mathsf{Preruns}(\mathfrak{P})$. Note that if two asynchronous programs $\mathfrak{P}$ and $\mathfrak{P}'$ have the same $D$ and $\Sigma$, then $\mathsf{Preruns}(\mathfrak{P}) = \mathsf{Preruns}(\mathfrak{P}')$. The *length*, denoted $|\rho|$, of a finite prerun $\rho$ is the number of configurations in $\rho$. The $i^{th}$ configuration of a prerun $\rho$ will be denoted $\rho(i)$.

We define an order $\trianglelefteq$ on preruns as follows: For preruns $\rho = (e_0, \mathbf{n}_0), \sigma_1, (e_1, \mathbf{n}_1), \sigma_2, \ldots$ and $\rho' = (e'_0, \mathbf{n}'_0), \sigma'_1, (e'_1, \mathbf{n}'_1), \sigma'_2, \ldots$, we define $\rho \trianglelefteq \rho'$ if $|\rho| = |\rho'|$ and $e_i = e'_i, \sigma_i = \sigma'_i$ and $\mathbf{n}_i \preceq \mathbf{n}'_i$ for each $i \geq 0$. The *downclosure* $\downarrow R$ of a set $R$ of preruns of $\mathfrak{P}$ is defined as $\downarrow R = \{\rho \in \mathsf{Preruns}(\mathfrak{P}) \mid \exists \rho' \in R.\ \rho \trianglelefteq \rho'\}$.

A *run* of an AP $\mathfrak{P} = (D, \Sigma, (L_c)_{c\in\mathfrak{C}}, d_0, \mathbf{m}_0)$ is a prerun $\rho = (d_0, \mathbf{m}_0), \sigma_1, (d_1, \mathbf{m}_1), \sigma_2, \ldots$ starting with the initial configuration $(d_0, \mathbf{m}_0)$, where for each $i \geq 0$, we have $(d_i, \mathbf{m}_i) \xrightarrow{\sigma_{i+1}} (d_{i+1}, \mathbf{m}_{i+1})$. The set of runs of $\mathfrak{P}$ is denoted $\mathsf{Runs}(\mathfrak{P})$ and $\downarrow\mathsf{Runs}(\mathfrak{P})$ is its downclosure with respect to $\trianglelefteq$.

An infinite run $c_0 \xrightarrow{\sigma_0} c_1 \xrightarrow{\sigma_1} \ldots$ is *fair* if for all $i \geq 0$, if $\sigma \in c_i.\mathbf{m}$ then there is some $j \geq i$ such that $c_j \xrightarrow{\sigma} c_{j+1}$. That is, whenever an instance of a handler is posted, some instance of the handler is executed later. Fairness does not preclude that a specific instance of a handler is never executed. An infinite fair run *starves* handler $\sigma$ if there exists an index $J \geq 0$ such that for each $j \geq J$, we have (i) $c_j.\mathbf{m}(\sigma) \geq 1$ and (ii) whenever $c_j \xrightarrow{\sigma} c_{j+1}$, we have $c_j.\mathbf{m}(\sigma) \geq 2$. In this case, even if the run is fair, a specific instance of $\sigma$ may never be executed.

Now we give the definitions of the various decision problems.

**Definition 2 (Properties of finite runs).** *The* **Safety (Global state reachability)** *problem asks, given an asynchronous program $\mathfrak{P}$ and a global state $d_f \in D$, is there a reachable configuration $c$ such that $c.d = d_f$? If so, $d_f$ is said to be* reachable *(in $\mathfrak{P}$) and* unreachable *otherwise. The* **Boundedness (of the task buffer)** *problem asks, given an asynchronous program $\mathfrak{P}$, is there an $N \in \mathbb{N}$ such that for every reachable configuration $c$, we have $|c.\mathbf{m}| \leq N$? If so, the asynchronous program $\mathfrak{P}$ is* bounded*; otherwise it is* unbounded. *The* **Configuration reachability** *problem asks, given an asynchronous program $\mathfrak{P}$ and a configuration $c$, is $c$ reachable?*

**Definition 3 (Properties of infinite runs).** *All the following problems take as input an asynchronous program $\mathfrak{P}$. The* **Termination** *problem asks if all runs of $\mathfrak{P}$ are finite. The* **Fair Non-termination** *problem asks if $\mathfrak{P}$ has some fair infinite run. The* **Fair Starvation** *problem asks if $\mathfrak{P}$ has some fair run that starves some handler.*

Our main result in this section shows that many properties of an asynchronous program $\mathfrak{P}$ only depend on the downclosure $\downarrow\mathsf{Runs}(\mathfrak{P})$ of the set $\mathsf{Runs}(\mathfrak{P})$ of runs of the program $\mathfrak{P}$. The proof is by induction on the length of runs. For any $\mathsf{AP}$ $\mathfrak{P} = (D, \Sigma, (L_c)_{c \in \mathfrak{C}}, d_0, \mathbf{m}_0)$, we define the $\mathsf{AP}$ $\downarrow\mathfrak{P} = (D, \Sigma, (\downarrow L_c)_{c \in \mathfrak{C}}, d_0, \mathbf{m}_0)$, where $\downarrow L_c$ is the downclosure of the language $L_c$ under the subword order.

**Proposition 1.** *Let $\mathfrak{P} = (D, \Sigma, (L_c)_{c \in \mathfrak{C}}, d_0, \mathbf{m}_0)$ be an asynchronous program. Then $\downarrow\mathsf{Runs}(\downarrow\mathfrak{P}) = \downarrow\mathsf{Runs}(\mathfrak{P})$. In particular, the following holds. (1) For every $d \in D$, $\mathfrak{P}$ can reach $d$ if and only if $\downarrow\mathfrak{P}$ can reach $d$. (2) $\mathfrak{P}$ is terminating if and only if $\downarrow\mathfrak{P}$ is terminating. (3) $\mathfrak{P}$ is bounded if and only if $\downarrow\mathfrak{P}$ is bounded.*

Intuitively, safety, termination, and boundedness is preserved when the multiset of pending handler instances is "lossy": posted handlers can get lost. This corresponds to these handlers never being scheduled by the scheduler. However, if a run demonstrates reachability of a global state, or non-termination, or unboundedness, in the lossy version, it corresponds also to a run in the original problem (and conversely). In contrast, simple examples show that configuration reachability, fair termination, and fair non-starvation properties are not preserved under downclosures.

## 4 General Decidability Results

In this section, we characterize those full trios $\mathcal{C}$ for which particular problems for asynchronous programs over $\mathcal{C}$ are decidable. Our decision procedures will use the following theorem, summarizing the results from [12], as a subprocedure.

**Theorem 1 ([12]).** *Safety, boundedness, configuration reachability, termination, fair non-termination, and fair non-starvation are decidable for asynchronous programs over regular languages.*

### 4.1 Safety and termination

Our first main result concerns the problems of safety and termination.

**Theorem 2.** *Let $\mathcal{C}$ be a full trio. The following are equivalent:*

*(i) Safety is decidable for asynchronous programs over $\mathcal{C}$.*
*(ii) Termination is decidable for asynchronous programs over $\mathcal{C}$.*
*(iii) Emptiness is decidable for $\mathcal{C}$.*

We begin with "(i)⇒(iii)". Let $K \subseteq \Sigma^*$ be given. We construct $\mathfrak{P} = (D, \Sigma, (L_c)_{c\in\mathfrak{C}}, d_0, \mathbf{m}_0)$ such that $\mathbf{m}_0 = [\![\sigma]\!]$, $D = \{d_0, d_1\}$, $L_{d_0,\sigma,d_1} = K$ and $L_c = \emptyset$ for $c \neq (d_0, \sigma, d_1)$. We see that $\mathfrak{P}$ can reach $d_1$ iff $K$ is non-empty. Next we show "(ii)⇒(iii)". Consider the alphabet $\Gamma = (\Sigma \cup \{\varepsilon\}) \times \{0, 1\}$ and the homomorphisms $g\colon \Gamma^* \to \Sigma^*$ and $h\colon \Gamma^* \to \{\sigma\}^*$, where for $x \in \Sigma \cup \{\varepsilon\}$, we have $g((x, i)) = x$ for $i \in \{0, 1\}$, $h((x, 1)) = \sigma$, and $h((x, 0)) = \varepsilon$. If $R \subseteq \Gamma^*$ is the regular set of words in which exactly one position belongs to the subalphabet $(\Sigma \cup \{\varepsilon\}) \times \{1\}$, then the language $K' := h(g^{-1}(K) \cap R)$ belongs to $\mathcal{C}$. Note that $K'$ is $\emptyset$ or $\{\sigma\}$, depending on whether $K$ is empty or not. We construct $\mathfrak{P} = (D, \Sigma, (L_c)_{c\in\mathfrak{C}}, d_0, \mathbf{m}_0)$ with $D = \{d_0\}$, $\mathbf{m}_0 = [\![\sigma]\!]$, $L_{d_0,\sigma,d_0} = K'$ and all languages $L_c = \emptyset$ for $c \neq (d_0, \sigma, d_0)$. Then $\mathfrak{P}$ is terminating iff $K$ is empty.

To prove "(iii)⇒(i)", we design an algorithm deciding safety assuming decidability of emptiness. Given asynchronous program $\mathfrak{P}$ and state $d$ as input, the algorithm consists of two semi-decision procedures: one which searches for a run of $\mathfrak{P}$ reaching the state $d$, and the second which enumerates regular overapproximations $\mathfrak{P}'$ of $\mathfrak{P}$ and checks the safety of $\mathfrak{P}'$ using Theorem 1. Each $\mathfrak{P}'$ consists of a regular language $A_c$ overapproximating $L_c$ for each context $c$ of $\mathfrak{P}$. We use decidability of emptiness to check that $L_c \cap (\Sigma^* \setminus A_c) = \emptyset$ to ensure that $\mathfrak{P}'$ is indeed an overapproximation.

The algorithm clearly gives a correct answer if it terminates. Hence, we only have to argue that it always does terminate. Of course, if $d$ is reachable, the first semi-decision procedure will terminate. In the other case, termination is due to the regularity of downclosures: if $d$ is not reachable in $\mathfrak{P}$, then Proposition 1 tells us that $\downarrow\mathfrak{P}$ cannot reach $d$ either. But $\downarrow\mathfrak{P}$ is an asynchronous program over regular languages; this means there exists a safe regular overapproximation and the second semi-decision procedure terminates.

Like the algorithm for safety, the algorithm for termination consists of two semi-decision procedures. By standard well-quasi-ordering arguments, an infinite run of an asynchronous program $\mathfrak{P}$ is witnessed by a finite self-covering run. The first semi-decision procedure enumerates finite self-covering runs (trying to show non-termination). The second procedure enumerates regular asynchronous programs $\mathfrak{P}'$ that overapproximate $\mathfrak{P}$. As before, to check termination of $\mathfrak{P}'$, it applies the procedure from Theorem 1. Clearly, the algorithm's answer is always correct. Moreover, it gives an answer for every input. If $\mathfrak{P}$ does not terminate, it will find a self-covering sequence. If $\mathfrak{P}$ does terminate, then Proposition 1 tells us that $\downarrow\mathfrak{P}$ is a terminating finite-state overapproximation. This implies that the second procedure will terminate in that case.

Let us point out a particular example. The class $\mathcal{L}$ of languages of lossy channel systems is defined like the class of languages of WSTS with upward-closed sets of accepting configurations as in [13], except that we only consider lossy channel systems [1] instead of arbitrary Well-Structured Transition Systems (WSTS). Then $\mathcal{L}$ forms a full trio with decidable emptiness. Although downclosures of lossy channel languages are not effectively computable (an easy consequence of [27]), our algorithm employs Theorem 2 to decide safety and termination.

### 4.2 Boundedness

**Theorem 3.** *Let $\mathcal{C}$ be a full trio. The following are equivalent:*

*(i) Boundedness is decidable for asynchronous programs over $\mathcal{C}$.*
*(ii) Finiteness is decidable for $\mathcal{C}$.*

Clearly, the construction for "(i)⇒(iii)" of Theorem 2 also works for "(i)⇒(ii)": $\mathfrak{P}$ is unbounded iff $K$ is infinite.

For the converse, we first note that if finiteness is decidable for $\mathcal{C}$ then so is emptiness. Given $L \subseteq \Sigma^*$ from $\mathcal{C}$, consider the homomorphism $h\colon (\Sigma \cup \{\lambda\})^* \to \Sigma^*$ with $h(\mathtt{a}) = \mathtt{a}$ for every $\mathtt{a} \in \Sigma$ and $h(\lambda) = \varepsilon$. Then $h^{-1}(L)$ belongs to $\mathcal{C}$ and $h^{-1}(L)$ is finite if and only if $L$ is empty: in the inverse homomorphism, $\lambda$ can be arbitrarily inserted in any word. By Theorem 2, this implies that we can also decide safety. As a consequence of considering only full trios, it is easy to see that the problem of *context reachability* reduces to safety: a context $\hat{c} = (\hat{d}, \hat{\sigma}, \hat{d}') \in \mathfrak{C}$ is *reachable in* $\mathfrak{P}$ if there is a reachable configuration $(\hat{d}, \mathbf{m})$ in $\mathfrak{P}$ with $\mathbf{m}(\hat{\sigma}) \geq 1$.

We now explain our algorithm for deciding boundedness of a given aysnchronous program $\mathfrak{P} = (D, \Sigma, (L_c)_{c\in\mathfrak{C}}, d_0, \mathbf{m}_0)$. For every context $c$, we first check if $L_c$ is infinite (feasible by assumption). This paritions the set of contexts of $\mathfrak{P}$ into sets $I$ and $F$ which are the contexts for which the corresponding language $L_c$ is infinite and finite respectively. If any context in $I$ is reachable, then $\mathfrak{P}$ is unbounded. Otherwise, all the reachable contexts have a finite language. For every finite language $L_c$ for some $c \in F$, we explicitly find all the members of $L_c$. This is possible because any finite set $A$ can be checked with $L_c$ for equality. $L_c \subseteq A$ can be checked by testing whether $L_c \cap (\Sigma^* \setminus A) = \emptyset$ and $L_c \cap (\Sigma^* \setminus A)$ effectively belongs to $\mathcal{C}$. On the other hand, checking $A \subseteq L_c$ just means checking whether $L_c \cap \{w\} \neq \emptyset$ for each $w \in A$, which can be done the same way. We can now construct asynchronous program $\mathfrak{P}'$ which replaces all languages for contexts in $I$ by $\emptyset$ and replaces those corresponding to $F$ by the explicit description. Clearly $\mathfrak{P}'$ is bounded iff $\mathfrak{P}$ is bounded (since no contexts from $I$ are reachable) and the former can be decided by Theorem 1.

We observe that boundedness is strictly harder than safety or termination: There are full trios for which emptiness is decidable, but finiteness is undecidable, such as the languages of reset vector addition systems [11] (see [32] for a definition of the language class) and languages of lossy channel systems.

### 4.3 Configuration reachability and liveness properties

Theorems 2 and 3 completely characterize for which full trios safety, termination, and boundedness are decidable. We turn to configuration reachability, fair termination, and fair starvation. We suspect that it is unlikely that there is a simple characterization of those language classes for which the latter problems are decidable. However, we show that they are decidable for a limited range of infinite-state systems. To this end, we prove that decidability of any of these problems implies decidability of the others as well, and also implies the decidability of a simple combinatorial problem that is known to be undecidable for many expressive classes of languages.

Let $Z \subseteq \{\mathtt{a}, \mathtt{b}\}^*$ be the language $Z = \{w \in \{\mathtt{a}, \mathtt{b}\}^* \mid |w|_{\mathtt{a}} = |w|_{\mathtt{b}}\}$. The *$Z$-intersection problem* for a language class $\mathcal{C}$ asks, given a language $K \subseteq \{\mathtt{a}, \mathtt{b}\}^*$ from $\mathcal{C}$, whether $K \cap Z \neq \emptyset$. Informally, $Z$ is the language of all words with an equal number of $\mathtt{a}$s and $\mathtt{b}$s and the $Z$-intersection problem asks if there is a word in $K$ with an equal number of $\mathtt{a}$s and $\mathtt{b}$s.

**Theorem 4.** *Let $\mathcal{C}$ be a full trio. The following statements are equivalent:*

*(i) Configuration reachability is decidable for asynchronous programs over $\mathcal{C}$.*
*(ii) Fair termination is decidable for asynchronous programs over $\mathcal{C}$.*
*(iii) Fair starvation is decidable for asynchronous programs over $\mathcal{C}$.*

*Moreover, if decidability holds, then $Z$-intersection is decidable for $\mathcal{C}$.*

We prove Theorem 4 by providing reductions among the three problems and showing that $Z$-intersection reduces to configuration reachability. We use diagrams similar to automata to describe asynchronous programs. Here, circles represent global states of the program and we draw an edge $\textcircled{d} \xrightarrow{\sigma|L} \textcircled{d'}$ in case we have $L_{d,\sigma,d'} = L$ in our asynchronous program $\mathfrak{P}$. Furthermore, we have $L_{d,\sigma,d'} = \emptyset$ whenever there is no edge that specifies otherwise. To simplify notation, we draw an edge $d \xrightarrow{w|L} d'$ in an asynchronous program for a word $w \in \Sigma^*$, $w = \sigma_1 \ldots \sigma_n$ with $\sigma_1, \ldots, \sigma_n \in \Sigma$, to symbolize a sequence of states

$$\textcircled{d} \xrightarrow{\sigma_1|\{\varepsilon\}} \textcircled{2} \xrightarrow{\sigma_2|\{\varepsilon\}} \cdots \xrightarrow{\sigma_{n-1}|\{\varepsilon\}} \textcircled{n} \xrightarrow{\sigma_n|L} \textcircled{d'}$$

which removes $[\![\sigma_1, \ldots, \sigma_n]\!]$ from the task buffer and posts a multiset of handlers specified by $L$.

*Proof of "(ii)⇒(i)"* Given an asynchronous program $\mathfrak{P} = (D, \Sigma, (L_c)_{c\in\mathfrak{C}}, d_0, \mathbf{m}_0)$ and a configuration $(d_f, \mathbf{m}_f) \in D \times \mathbb{M}[\Sigma]$, we construct asynchronous program $\mathfrak{P}'$ as follows. Let $\mathtt{z}$ be a fresh letter and let $\mathbf{m}_f = [\![\sigma_1, \ldots, \sigma_n]\!]$. We obtain $\mathfrak{P}'$ from $\mathfrak{P}$ by adding a new state $d'_f$ and including the following edges:

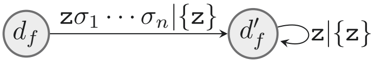

Starting from $(d_0, \mathbf{m}_0 \oplus [\![\mathtt{z}]\!])$, the program $\mathfrak{P}'$ has a fair infinite run iff $(d_f, \mathbf{m}_f)$ is reachable in $\mathfrak{P}$. The 'if' direction is obvious. Conversely, $\mathtt{z}$ has to be executed in any fair run $\rho$ of $\mathfrak{P}'$ which implies that $d'_f$ is reached by $\mathfrak{P}'$ in $\rho$. Since only $\mathtt{z}$ can be executed at $d'_f$ in $\rho$, this means that the multiset is exactly $\mathbf{m}_f$ when $d_f$ is reached during $\rho$. Clearly this initial segment of $\rho$ corresponds to a run of $\mathfrak{P}$ which reaches the target configuration.

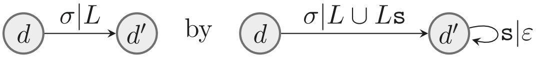

*Proof of "(iii)⇒(ii)"* We construct $\mathfrak{P}' = (D, \Sigma', (L'_c)_{c\in\mathfrak{C}'}, d_0, \mathbf{m}'_0)$ given $\mathfrak{P} = (D, \Sigma, (L_c)_{c\in\mathfrak{C}}, d_0, \mathbf{m}_0)$ over $\mathcal{C}$ as follows. Let $\Sigma' = \Sigma \cup \{\mathtt{s}\}$, where $\mathtt{s}$ is a fresh handler. Replace each edge at every state $d \in D$. Moreover, we set $\mathbf{m}'_0 = \mathbf{m}_0 \oplus [\![\mathtt{s}, \mathtt{s}]\!]$. Then $\mathfrak{P}'$ has an infinite fair run that starves some handler if and only if $\mathfrak{P}$ has an infinite fair run. From an infinite fair run $\rho$ of $\mathfrak{P}$, we obtain an infinite fair run of $\mathfrak{P}'$ which starves $\mathtt{s}$, by producing $\mathtt{s}$ while simulating $\rho$ and consuming it in the loop. Conversely, from an infinite fair run $\rho'$ of $\mathfrak{P}'$ which starves some $\tau$, we obtain an infinite fair run $\rho$ of $\mathfrak{P}$ by omitting all productions and consumptions of $\mathtt{s}$ and removing two extra instances of $\mathtt{s}$ from all configurations.

*Proof of "(i)⇒(iii)"* From $\mathfrak{P} = (D, \Sigma, (L_c)_{c\in\mathfrak{C}}, d_0, \mathbf{m}_0)$ over $\mathcal{C}$, for each subset $\Gamma \subseteq \Sigma$ and $\tau \in \Sigma$, we construct an asynchronous program $\mathfrak{P}_{\Gamma,\tau} = (D', \Sigma', (L_c)_{c\in\mathfrak{C}'}, d'_0, \mathbf{m}'_0)$ over $\mathcal{C}$ such that a particular configuration is reachable in $\mathfrak{P}_{\Gamma,\tau}$ if and only if $\mathfrak{P}$ has a fair infinite run $\rho_{\Gamma,\tau}$, where $\Gamma$ is the set of handlers that is executed infinitely often in $\rho_{\Gamma,\tau}$ and $\rho_{\Gamma,\tau}$ starves $\tau$. Since there are only finitely many choices for $\Gamma$ and $\tau$, decidability of configuration reachability implies decidability of fair starvation. The idea is that run $\rho_{\Gamma,\tau}$ exists if and only if there exists a run

$$(d_0, \mathbf{m}_0) \xrightarrow{\sigma_1} \cdots \xrightarrow{\sigma_n} (d_n, \mathbf{m}_n) = (e_0, \mathbf{n}_0) \xrightarrow{\gamma_1} (e_1, \mathbf{n}_1) \xrightarrow{\gamma_2} \cdots \xrightarrow{\gamma_k} (e_k, \mathbf{n}_k), \quad (1)$$

where $\bigcup_{i=1}^{k}\{\gamma_i\} = \Gamma$, for each $1 \le i \le k$ $\mathbf{n}_i \in \mathbb{M}[\Gamma]$, $\mathbf{m}_n \preceq \mathbf{n}_k$, and for each $i \in \{1, \ldots, k\}$ with $\gamma_i = \tau$, we have $\mathbf{n}_{i-1}(\tau) \ge 2$. In such a run, we call $(d_0, \mathbf{m}_0) \xrightarrow{\sigma_1} \cdots \xrightarrow{\sigma_n} (d_n, \mathbf{m}_n)$ its *first phase* and $(e_0, \mathbf{n}_0) \xrightarrow{\gamma_1} \cdots \xrightarrow{\gamma_k} (e_k, \mathbf{n}_k)$ its *second phase.*

Let us explain how $\mathfrak{P}_{\Gamma,\tau}$ reflects the existence of a run as in Eq. (1). The set $\Sigma'$ of handlers of $\mathfrak{P}_{\Gamma,\tau}$ includes $\Sigma$, $\bar{\Sigma}$ and $\hat{\Sigma}$, where $\bar{\Sigma} = \{\bar{\sigma} \mid \sigma \in \Sigma\}$ and $\hat{\Sigma} = \{\hat{\sigma} \mid \sigma \in \Sigma\}$ are disjoint copies of $\Sigma$. This means, a multiset $\mathbb{M}[\Sigma']$ contains multisets $\mathbf{m}' = \mathbf{m} \oplus \bar{\mathbf{m}} \oplus \hat{\mathbf{m}}$ with $\mathbf{m} \in \mathbb{M}[\Sigma]$, $\bar{\mathbf{m}} \in \mathbb{M}[\bar{\Sigma}]$, and $\hat{\mathbf{m}} \in \mathbb{M}[\hat{\Sigma}]$. A run of $\mathfrak{P}_{\Gamma,\tau}$ simulates the two phases of $\rho$. While simulating the first phase, $\mathfrak{P}_{\Gamma,\tau}$ keeps two copies of the task buffer, $\mathbf{m}$ and $\bar{\mathbf{m}}$. The copying is easily accomplished by a homomorphism with $\sigma \mapsto \sigma\bar{\sigma}$ for each $\sigma \in \Sigma$. At some point, $\mathfrak{P}_{\Gamma,\tau}$ switches into simulating the second phase. There, $\bar{\mathbf{m}}$ remains unchanged, so that it stores the value of $\mathbf{m}_n$ in Eq. (1) and can be used in the end to make sure that $\mathbf{m}_n \preceq \mathbf{n}_k$.

Hence, in the second phase, $\mathfrak{P}_{\Gamma,\tau}$ works, like $\mathfrak{P}$, only with $\Sigma$. However, whenever a handler $\sigma \in \Sigma$ is executed, it also produces a task $\hat{\sigma}$. These handlers are used at the end to make sure that every $\gamma \in \Gamma$ has been executed at least once in the second phase. Also, whenever $\tau$ is executed, $\mathfrak{P}_{\Gamma,\tau}$ checks that at least two instances of $\tau$ are present in the task buffer, thereby ensuring that $\tau$ is starved.

In the end, a distinguished final state allows $\mathfrak{P}_{\Gamma,\tau}$ to execute handlers in $\Gamma$ and $\bar{\Gamma}$ simultaneously to make sure that $\mathbf{m}_n \preceq \mathbf{n}_k$. In its final state, $\mathfrak{P}_{\Gamma,\tau}$ can execute handlers $\hat{\gamma} \in \hat{\Gamma}$ and $\gamma \in \Gamma$ (without creating new handlers). In the final configuration, there can be no $\hat{\sigma}$ with $\sigma \in \Sigma \setminus \Gamma$, and there has to be exactly one $\hat{\gamma}$ for each $\gamma \in \Gamma$. This guarantees that (i) each handler in $\Gamma$ is executed at least once during the second phase, (ii) every handler executed in the second phase is from $\Gamma$, and (iii) $\mathbf{m}_n$ contains only handlers from $\Gamma$ (because handlers from $\bar{\Sigma}$ cannot be executed in the second phase).

*Decidability of Z-intersection* To complete the proof of Theorem 4, we reduce $Z$-intersection to configuration reachability. Given $K \subseteq \{\mathtt{a}, \mathtt{b}\}^*$ from $\mathcal{C}$, we construct the asynchronous program $\mathfrak{P} = (D, \Sigma, (L_c)_{c\in\mathfrak{C}}, d_0, \mathbf{m}_0)$ over $\mathcal{C}$ where $D = \{d_0, 0, 1\}$, $\Sigma = \{\mathtt{a}, \mathtt{b}, \mathtt{c}\}$, by including the following edges:

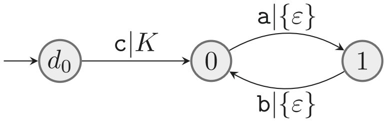

The initial task buffer is $\mathbf{m}_0 = [\![\mathtt{c}]\!]$. Then clearly, the configuration $(0, [\![]\!])$ is reachable in $\mathfrak{P}$ if and only if $K \cap Z \neq \emptyset$.

Theorem 4 is useful in the contrapositive to show undecidability. For example, one can show undecidability of $Z$-intersection for languages of lossy channel systems (see Section 4.1): One expresses reachability in a non-lossy FIFO system by making sure that the numbers of enqueue- and dequeue-operations match. Thus, for asynchronous programs over lossy channel systems, the problems of Theorem 4 are undecidable. We also use Theorem 4 in Section 5 to conclude undecidability for higher-order asynchronous programs, already at order 2.

## 5 Higher-Order Asynchronous Programs

We apply our general decidability results to asynchronous programs over (deterministic) higher-order recursion schemes (HORS). Kobayashi [21] has shown how higher-order functional programs can be modeled using HORS. In his setting, a program contains instructions that access certain resources. For Kobayashi, the path language of the HORS is the set of possible sequences of instructions. For us, the input program contains `post` instructions and we translate higher-order programs with `post` instructions into a HORS whose path language is used as the language of handlers.
We recall some definitions from [21]. The set of *types* is defined by the grammar $A ::= \mathsf{o} \mid A \to A$. The *order* $\mathrm{ord}(A)$ of a type $A$ is inductively defined as $\mathrm{ord}(\mathsf{o}) = 0$ and $\mathrm{ord}(A \to B) := \max(\mathrm{ord}(A) + 1, \mathrm{ord}(B))$. The *arity* of a type is inductively defined by $\mathrm{arity}(\mathsf{o}) = 0$ and $\mathrm{arity}(A \to B) = \mathrm{arity}(B) + 1$. We assume a countably infinite set Var of typed variables $x : A$. For a set $\Theta$ of typed symbols, the set $\tilde{\Theta}$ of *terms* generated from $\Theta$ is the least set which contains $\Theta$ such that whenever $s : A \to B$ and $t : A$ belong to $\tilde{\Theta}$, then also $s\,t : B$ belongs to $\tilde{\Theta}$. By convention the type $\mathsf{o} \to \ldots (\mathsf{o} \to (\mathsf{o} \to \mathsf{o}))$ is written $\mathsf{o} \to \ldots \to \mathsf{o} \to \mathsf{o}$ and the term $((t_1 t_2) t_3 \cdots) t_n$ is written $t_1 t_2 \cdots t_n$. We write $\bar{x}$ for a sequence $(x_1, x_2, \ldots, x_n)$ of variables.

A higher-order recursion scheme (HORS) is a tuple $\mathscr{S} = (\Sigma, \mathcal{N}, \mathcal{R}, S)$ where $\Sigma$ is a set of typed *terminal* symbols of types of order 0 or 1, $\mathcal{N}$ is a set of typed *non-terminal* symbols (disjoint from terminal symbols), $S : \mathsf{o}$ is the start non-terminal symbol and $\mathcal{R}$ is a set of rewrite rules $F x_1 x_2 \cdots x_n \twoheadrightarrow t$ where $F : A_1 \to \cdots \to A_n \to \mathsf{o}$ is a non-terminal in $\mathcal{N}$, $x_i : A_i$ for all $i$ are variables and $t : \mathsf{o}$ is a term generated from $\Sigma \cup \mathcal{N} \cup \mathrm{Var}$. The order of a HORS is the maximum order of a non-terminal symbol. We define a rewrite relation $\twoheadrightarrow$ on terms over $\Sigma \cup \mathcal{N}$ as follows: $F\bar{a} \twoheadrightarrow t[\bar{x}/\bar{a}]$ if $F\bar{x} \twoheadrightarrow t \in \mathcal{R}$, and if $t \twoheadrightarrow t'$ then $ts \twoheadrightarrow t's$ and $st \twoheadrightarrow st'$. The reflexive, transitive closure of $\twoheadrightarrow$ is denoted $\twoheadrightarrow^*$. A *sentential form* $t$ of $\mathscr{S}$ is a term over $\Sigma \cup \mathcal{N}$ such that $S \twoheadrightarrow^* t$.

If $N$ is the maximum arity of a symbol in $\Sigma$, then a (possibly infinite) tree over $\Sigma$ is a partial function *tr* from $\{0, 1, \ldots, N-1\}^*$ to $\Sigma$ that fulfills the following conditions: $\varepsilon \in \mathrm{dom}(tr)$, $\mathrm{dom}(tr)$ is closed under prefixes, and if $tr(w) = a$ and $\mathrm{arity}(a) = k$ then $\{j \mid wj \in \mathrm{dom}(tr)\} = \{0, 1, \ldots, k-1\}$.

A *deterministic* HORS is one where there is exactly one rule of the form $F x_1 x_2 \cdots x_n \to t$ for every non-terminal $F$. Following [21], we show how a deterministic HORS can be used to represent a higher-order pushdown language arising from a higher-order functional program.

Sentential forms can be seen as ranked trees over $\Sigma \cup \mathcal{N} \cup \mathrm{Var}$. A sequence $\Pi$ over $\{0, 1, \ldots, n-1\}$ is a *path* of *tr* if every finite prefix of $\Pi \in \mathrm{dom}(tr)$. The set of paths in a tree *tr* will be denoted $\mathsf{Paths}(tr)$. Note that we are only interested in finite paths in our context. Associated with any path $\Pi = n_1, n_2, \ldots, n_k$ is the word $w_\Pi = tr(n_1) tr(n_1 n_2) \cdots tr(n_1 n_2 \cdots n_k)$. Let $\Sigma_1 := \{a \in \Sigma \mid \mathrm{arity}(a) = 1\}$. The *path language* $\mathcal{L}_{\mathsf{p}}(\mathscr{S})$ of a deterministic HORS $\mathscr{S}$ is defined as $\{\mathrm{Proj}_{\Sigma_1}(w_\Pi) \mid \Pi \in \mathsf{Paths}(\mathcal{T}_{\mathscr{S}})\}$. The *tree language* $\mathcal{L}_{\mathsf{t}}(\mathscr{S})$ associated with a HORS is the set of finite trees over $\Sigma$ generated by $\mathscr{S}$.
The deterministic HORS corresponding to the higher-order function `s3` from Figure 1 is given by $\mathscr{S} = (\Sigma, \mathcal{N}, \mathcal{R}, S)$, where

$$\begin{aligned}
\Sigma &= \{\mathtt{br} : \mathsf{o} \to \mathsf{o} \to \mathsf{o}, \mathtt{c}, \mathtt{d}, \mathtt{f} : \mathsf{o} \to \mathsf{o}, \mathtt{e} : \mathsf{o}\} \\
\mathcal{N} &= \{S : \mathsf{o}, F : (\mathsf{o} \to \mathsf{o}) \to \mathsf{o} \to \mathsf{o}, H : (\mathsf{o} \to \mathsf{o}) \to \mathsf{o} \to \mathsf{o}, I : \mathsf{o} \to \mathsf{o}\} \\
\mathcal{R} &= \{S \twoheadrightarrow F\ I\ \mathtt{e}, I\ x \twoheadrightarrow x, F\ G\ x \twoheadrightarrow \mathtt{br}(F\ (H\ G)\ (\mathtt{f}x))\ (G\ x), \\
&\qquad H\ G\ x \twoheadrightarrow \mathtt{c}(G(\mathtt{d}x))\}
\end{aligned}$$

The path language $\mathcal{L}_{\mathsf{p}}(\mathscr{S}) = \{\mathtt{c}^n\mathtt{d}^n\mathtt{f}^n \mid n \geq 0\}$. To see this, apply the reduction rules to get the value tree $\mathcal{T}_{\mathscr{S}}$ shown on the right:

$$\begin{aligned} S &\twoheadrightarrow F\ I\ \mathtt{e} \twoheadrightarrow \mathtt{br}\ (F\ (HI)\ (\mathtt{fe}))\ (I\mathtt{e}) \\ &\twoheadrightarrow \mathtt{br}\ (F\ (HI)\ (\mathtt{fe}))\ \mathtt{e} \\ &\twoheadrightarrow \mathtt{br}\ (\mathtt{br}\ (F\ (H^2I)\ (\mathtt{f}^2e))\ (HI)(\mathtt{fe}))\ \mathtt{e} \\ &\twoheadrightarrow \mathtt{br}\ (\mathtt{br}\ (F\ (H^2I)\ (\mathtt{f}^2\mathtt{e}))\ \mathtt{c}(I(\mathtt{dfe}))\ \mathtt{e} \\ &\twoheadrightarrow \mathtt{br}\ (\mathtt{br}\ (F\ (H^2I)\ (\mathtt{f}^2\mathtt{e}))\ \mathtt{cdfe})\ \mathtt{e} \\ &\twoheadrightarrow \cdots \end{aligned}$$

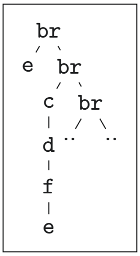

A HORS $\mathscr{S}$ is called a *word scheme* if it has exactly one nullary terminal symbol e and all other terminal symbols $\tilde{\Sigma}$ are of arity one. The *word language* $\mathcal{L}_{\mathsf{w}}(\mathscr{S}) \subseteq \tilde{\Sigma}^*$ defined by $\mathscr{S}$ is $\mathcal{L}_{\mathsf{w}}(\mathscr{S}) = \{a_1 a_2 \cdots a_n \mid (a_1(a_2 \cdots (a_n(\mathtt{e}))\cdots)) \in \mathcal{L}_{\mathsf{t}}(\mathscr{S})\}$. We denote by $\mathcal{H}$ the class of languages $\mathcal{L}_{\mathsf{w}}(\mathscr{S})$ that occur as the word language of a higher-order recursion scheme $\mathscr{S}$. Note that path languages and languages of word schemes are both word languages over the set $\tilde{\Sigma}$ of unary symbols considered as letters. They are connected by the following proposition.[2]

**Proposition 2.** *For every order-n* HORS $\mathscr{S} = (\Sigma, \mathcal{N}, S, \mathcal{R})$ *there exists an order-n word scheme* $\mathscr{S}' = (\Sigma', \mathcal{N}', S', \mathcal{R}')$ *such that* $\mathcal{L}_{\mathsf{p}}(\mathscr{S}) = \mathcal{L}_{\mathsf{w}}(\mathscr{S}')$.

A consequence of [21] and Prop. 2 is that the "post" language of higher-order functional programs can be modeled as the language of a word scheme. Hence, we define an *asynchronous program over* HORS as an asynchronous program over the language class $\mathcal{H}$ and we can use the following results on word schemes.

**Theorem 5.** HORS *and word schemes form effective full trios [7]. Emptiness [23] and finiteness [29] of order-n word schemes are* $(n-1)$-EXPTIME*-complete.*

Now Theorems 2 and 3, together with Proposition 2 imply the decidability results in Corollary 1. The undecidability result is a consequence of Theorem 4 and the undecidability of the $Z$-intersection problem for indexed languages or equivalently, order-2 pushdown automata as shown in [33]. Order-2 pushdown automata can be effectively turned into order-2 OI grammars [10], which in turn can be translated into order-2 word schemes [9]. See also [22, Theorem 4].

**Corollary 1.** *For asynchronous programs over* HORS*: (1) Safety, termination, and boundedness are decidable. (2) Configuration reachability, fair termination, and fair starvation are undecidable already at order-2.*

**A Direct Algorithm** We say that *downclosures are computable* for a language class $\mathcal{C}$ if for a given description of a language $L$ in $\mathcal{C}$, one can compute an automaton for the regular language $\downarrow L$. From Proposition 1 and Theorem 1,

[2] The models of HORS (used in model checking higher order programs [21]) and word schemes (used in language-theoretic exploration of downclosures [15,7]) are somewhat different. Thus, we show an explicit reduction between the two formalisms.

if one can compute downclosures for a language class, then one can avoid the enumerative approaches of Section 4 and get a "direct algorithm." The algorithm replaces each handler by its downclosure and then invokes the decision procedure summarized in Theorem 1. The direct algorithm for asynchronous programs over HORS relies on the recent breakthrough results on computing downclosures.

**Theorem 6 ([33,15,7]).** *Downclosures are effectively computable for $\mathcal{H}$.*

Unfortunately, current techniques for computing downclosures do not yet provide a complexity upper bound as we describe below. In [33], it was shown that in a full trio, downclosures are computable if and only if the *diagonal problem* for $\mathcal{C}$ is decidable. The latter asks, given a language $L \subseteq \Sigma^*$, whether for every $k \in \mathbb{N}$, there is a word $w \in L$ with $|w|_\sigma \geq k$ for every $\sigma \in \Sigma$. The diagonal problem was then shown to be decidable for higher-order pushdown automata [15] and then for word schemes [7]. The algorithm from [33] to compute downclosures using an oracle for the diagonal problem employs enumeration to compute a downclosure automaton, thus we have hidden the enumeration into the downclosure computation. We conjecture that downclosures can be computed in elementary time for word schemes of fixed order. This would imply an elementary time procedure for asynchronous programs over HORS of fixed order.

For handlers over context-free languages, given as PDAs, Ganty and Majumdar [12] show an EXPSPACE upper bound for safety, termination, and boundedness. Their algorithm constructs for each handler a polynomial-size Petri net with certain guarantees (forming so-called *adequate family of Petri nets*) that accepts a Parikh equivalent language. These Petri nets are then used to construct a larger Petri net, polynomial in the size of the asynchronous program and the adequate family of Petri nets, in which safety, termination, or boundedness can be phrased as a query decidable in EXPSPACE.

A natural question is whether a downclosure-based algorithm matches the same complexity. We can replace the Parikh-equivalent Petri nets of [12] with Petri nets recognizing the downclosure of a language. It is an easy consequence of Proposition 1 that the resulting Petri nets can be used in place of the adequate families of Petri nets in the procedures for safety, termination, and boundedness of [12]. Unfortunately, a finite automaton for $\downarrow L$ may require exponentially many states in the PDA [4], so a naive approach gives a 2EXPSPACE algorithm.

In the full version of this paper, we show that that for each context-free language $L$, one can construct in polynomial time a 1-bounded Petri net accepting $\downarrow L$. (Recall that a 1-bounded Petri net if every reachable marking has at most one token in each place.) When used in the construction of [12], this matches the EXPSPACE upper bound for safety, termination, and boundedness verification.

As a byproduct, we get a simple direct construction of a finite automaton for $\downarrow L$ when $L$ is given as a PDA. This is of independent interest because earlier constructions of $\downarrow L$ always start from a context-free grammar and produce (necessarily!) exponentially large NFAs [24,8,4]. The key observation is that the downclosure of the language of a PDA can be represented, after some simple modifications, as the language accepted by the PDA with a *bounded* stack.

## References

[1] Abdulla, P.A., Bouajjani, A., Jonsson, B.: On-the-fly analysis of systems with unbounded, lossy FIFO channels. In: Proceedings of the 10th International Conference on Computer Aided Verification (CAV 1998). pp. 305–318 (1998). 10.1007/BFb0028754

[2] Aho, A.V.: Indexed grammars - an extension of context-free grammars. J. ACM **15**(4), 647–671 (1968). 10.1145/321479.321488

[3] Atig, M.F., Bouajjani, A., Qadeer, S.: Context-bounded analysis for concurrent programs with dynamic creation of threads. In: Proceedings of TACAS 2009. pp. 107–123 (2009)

[4] Bachmeier, G., Luttenberger, M., Schlund, M.: Finite automata for the sub- and superword closure of CFLs: Descriptional and computational complexity. In: Proceedings of LATA 2015. pp. 473–485 (2015)

[5] Berstel, J.: Transductions and context-free languages. Teubner-Verlag (1979)

[6] Chadha, R., Viswanathan, M.: Decidability results for well-structured transition systems with auxiliary storage. In: CONCUR ’07: Proc. 18th Int. Conf. on Concurrency Theory. LNCS, vol. 4703, pp. 136–150. Springer (2007)

[7] Clemente, L., Parys, P., Salvati, S., Walukiewicz, I.: The diagonal problem for higher-order recursion schemes is decidable. In: Proceedings of the 31st Annual ACM/IEEE Symposium on Logic in Computer Science, LICS ’16, New York, NY, USA, July 5–8, 2016. pp. 96–105. ACM (2016). 10.1145/2933575.2934527

[8] Courcelle, B.: On constructing obstruction sets of words. Bulletin of the EATCS **44**, 178–186 (1991)

[9] Damm, W.: The IO-and OI-hierarchies. Theoretical Computer Science **20**(2), 95–207 (1982)

[10] Damm, W., Goerdt, A.: An automata-theoretical characterization of the OI-hierarchy. Information and Control **71**(1), 1–32 (1986)

[11] Dufourd, C., Finkel, A., Schnoebelen, P.: Reset nets between decidability and undecidability. In: Proceedings of ICALP 1998. pp. 103–115 (1998)

[12] Ganty, P., Majumdar, R.: Algorithmic verification of asynchronous programs. ACM Transactions on Programming Languages and Systems (TOPLAS) **34**(1), 6 (2012)

[13] Geeraerts, G., Raskin, J., Begin, L.V.: Well-structured languages. Acta Inf. **44**(3–4), 249–288 (2007). 10.1007/s00236-007-0050-3

[14] Greibach, S.A.: Remarks on blind and partially blind one-way multicounter machines. Theoretical Computer Science **7**(3), 311 – 324 (1978). 10.1016/0304-3975(78)90020-8

[15] Hague, M., Kochems, J., Ong, C.L.: Unboundedness and downward closures of higher-order pushdown automata. In: POPL 2016: Principles of Programming Languages. pp. 151–163. ACM (2016)

[16] Hague, M., Murawski, A.S., Ong, C.L., Serre, O.: Collapsible pushdown automata and recursion schemes. In: Proceedings of the Twenty-Third Annual IEEE Symposium on Logic in Computer Science, LICS 2008, 24–27 June 2008, Pittsburgh, PA, USA. pp. 452–461 (2008). 10.1109/LICS.2008.34

[17] Haines, L.H.: On free monoids partially ordered by embedding. Journal of Combinatorial Theory **6**(1), 94–98 (1969)

[18] Hopcroft, J.E., Motwani, R., Ullman, J.D.: Introduction to automata theory, languages, and computation, 3rd Edition. Pearson international edition, Addison-Wesley (2007)

[19] Jantzen, M.: On the hierarchy of Petri net languages. RAIRO - Theoretical Informatics and Applications - Informatique Théorique et Applications **13**(1), 19–30 (1979), http://www.numdam.org/item?id=ITA_1979__13_1_19_0

[20] Jhala, R., Majumdar, R.: Interprocedural analysis of asynchronous programs. In: POPL ’07: Proc. 34th ACM SIGACT-SIGPLAN Symp. on Principles of Programming Languages. pp. 339–350. ACM Press (2007)

[21] Kobayashi, N.: Types and higher-order recursion schemes for verification of higher-order programs. In: Proceedings of the 36th ACM SIGPLAN-SIGACT Symposium on Principles of Programming Languages, POPL 2009, Savannah, GA, USA, January 21–23, 2009. pp. 416–428 (2009). 10.1145/1480881.1480933

[22] Kobayashi, N.: Inclusion between the frontier language of a non-deterministic recursive program scheme and the Dyck language is undecidable. Theoretical Computer Science **777**, 409–416 (2019).

[23] Kobayashi, N., Ong, C.L.: Complexity of model checking recursion schemes for fragments of the modal mu-calculus. Logical Methods in Computer Science **7**(4) (2011)

[24] van Leeuwen, J.: Effective constructions in well-partially-ordered free monoids. Discrete Mathematics **21**(3), 237–252 (1978). 10.1016/0012-365X(78)90156-5

[25] Majumdar, R., Thinniyam, R.S., Zetzsche, G.: General decidability results for asynchronous shared-memory programs: Higher-order and beyond (2021), http://arxiv.org/abs/2101.08611

[26] Maslov, A.: The hierarchy of indexed languages of an arbitrary level. Doklady Akademii Nauk **217**(5), 1013–1016 (1974)

[27] Mayr, R.: Undecidable problems in unreliable computations. Theoretical Computer Science **297**(1–3), 337–354 (2003)

[28] Ong, L.: Higher-order model checking: An overview. In: 30th Annual ACM/IEEE Symposium on Logic in Computer Science, LICS 2015, Kyoto, Japan, July 6–10, 2015. pp. 1–15 (2015). 10.1109/LICS.2015.9

[29] Parys, P.: The complexity of the diagonal problem for recursion schemes. In: Proceedings of FSTTCS 2017. Leibniz International Proceedings in Informatics (LIPIcs), vol. 93, pp. 45:1–45:14 (2018)

[30] Sen, K., Viswanathan, M.: Model checking multithreaded programs with asynchronous atomic methods. In: CAV ’06: Proc. 18th Int. Conf. on Computer Aided Verification. LNCS, vol. 4144, pp. 300–314. Springer (2006)

[31] Sipser, M.: Introduction to the theory of computation. PWS Publishing Company (1997)

[32] Thinniyam, R.S., Zetzsche, G.: Regular separability and intersection emptiness are independent problems. In: Proceedings of FSTTCS 2019. Leibniz International Proceedings in Informatics (LIPIcs), vol. 150, pp. 51:1–51:15. Schloss Dagstuhl-Leibniz-Zentrum fuer Informatik, Dagstuhl, Germany (2019). 10.4230/LIPIcs.FSTTCS.2019.51

[33] Zetzsche, G.: An approach to computing downward closures. In: ICALP 2015. vol. 9135, pp. 440–451. Springer (2015), the undecidability of $$Z$$ intersection is shown in the full version: http://arxiv.org/abs/1503.01068

